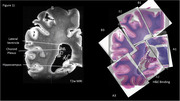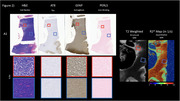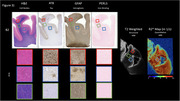# Post‐mortem MR imaging of tau pathology – a pilot study

**DOI:** 10.1002/alz.092362

**Published:** 2025-01-09

**Authors:** Vishaal Sumra, Shoshana Spring, Owen Botelho, Brian Nieman, John G Sled, Maria Carmela Tartaglia, Gabor G. Kovacs

**Affiliations:** ^1^ Institute of Medical Science, University of Toronto, Toronto, ON Canada; ^2^ Tanz Centre for Research in Neurodegenerative Diseases, University of Toronto, Toronto, ON Canada; ^3^ Mouse Imaging Centre ‐ Hospital for Sick Children, Toronto, ON Canada; ^4^ University of Toronto, Toronto, ON Canada; ^5^ Division of Neurology, Krembil Neuroscience Centre, Toronto Western Hospital, University Health Network Memory Clinic, Toronto, ON Canada; ^6^ Toronto Western Hospital, Tanz Centre for Research in Neurodegenerative Disease, Toronto, ON Canada; ^7^ Krembil Brain Institute, University Health Network (UHN), Toronto, ON Canada; ^8^ Division of Neurology, Toronto Western Hospital, University Health Network Memory Clinic, Toronto, ON Canada; ^9^ Department of Laboratory Medicine and Pathobiology, University of Toronto, Toronto, ON Canada; ^10^ Laboratory Medicine Program, University Health Network, Toronto, ON Canada; ^11^ The Edmond J. Safra Program in Parkinson’s Disease and Morton and Gloria Shulman Movement Disorders Clinic, Toronto, ON Canada; ^12^ Krembil Brain Institute, Toronto, ON Canada

## Abstract

**Background:**

In‐vivo detection of neuropathology is critical for early diagnosis of neurodegenerative diseases. Post‐mortem brain magnetic resonance imaging (MRI) of pathological protein inclusions could further our ability to detect them in vivo and correlate MRI parameters to histopathological substrates. In this post‐mortem study, we aimed to identify MRI correlates of neurodegenerative disease pathology in a brain with various forms of proteinopathies.

**Method:**

One large cortical section containing the temporal cortex, hippocampus and thalamus was used from an 82‐year‐old male patient, with chronic traumatic encephalopathy (CTE), Alzheimer’s disease (AD), TDP‐43, aging related tau astrogliosis (ARTAG) and cerebral amyloid angiopathy (CAA) pathologies. The post‐mortem tissue sample was scanned using high‐resolution Rapid Acquisition with Relaxation Enhancement (RARE) and multi‐gradient echo (MGE) sequences on an 11.7 Tesla MRI (Bruker, Germany). RARE scan parameters were as follows: field of view: 97.2 x 86.4 x 28.8 mm, rep time: 2 s, RARE factor: 8, echo time: 48 ms, echo spacing: 12 ms, 225 μm isotropic resolution. Tissue samples were subsequently sectioned (0.004 mm thick slices) and stained using AT8 – for Tau, GFAP – for astrogliosis and H&E/Luxol – for distinguishing gray (GM) and white matter (WM) and PERLS – for extracellular iron.

**Result:**

Post‐mortem MRI allowed for clear visualization of WM and GM structures. Decreased cell density in WM as visualised on H&E stained slices was associated with hyperintensities on T2w images and decreased measured R2*. AT8 immunoreactivity in GM and WM was associated with hyperintensities on T2w images and decreased measured R2*.

**Conclusion:**

High‐resolution MRI of post‐mortem tissue allowed for visualization of fine WM‐GM details, as well as hyperintensities in areas that potentially correspond to increased densities of tau pathology and decreased WM density. Further studies are needed to investigate the potential for high‐resolution post‐mortem MRI to visualize the pathological process in neurodegenerative diseases.